# Concomitant septal myectomy and mitral valve repair in a child with Noonan syndrome

**DOI:** 10.1002/ccr3.3531

**Published:** 2020-11-18

**Authors:** Toshio Doi, Daisuke Toritsuka, Akihiko Higashida, Shigeki Yokoyama, Kazuaki Fukahara, Naoki Yoshimura

**Affiliations:** ^1^ First Department of Surgery University of Toyama Toyama Japan

**Keywords:** hypertrophic obstructive cardiomyopathy, mitral valve regurgitation, mitral valve repair, Noonan syndrome, septal myectomy

## Abstract

We successfully performed concomitant septal myectomy and mitral valve repair in a child with Noonan syndrome.

## Graphical abstract

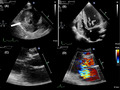

## 1 INTRODUCTION

A 7‐year‐old boy with Noonan syndrome presented to our hospital with symptomatic hypertrophic obstructive cardiomyopathy and mitral regurgitation associated with systolic anterior motion for surgery. Septal myectomy and mitral valve repair were performed. The left ventricular outflow tract was widened and systolic anterior motion and mitral regurgitation then improved.

Noonan syndrome is characterized by idiosyncratic facial features, skeletal abnormalities, mental retardation, and cardiac anomalies.^1^ Hypertrophic cardiomyopathy reportedly develops in approximately 16%‐22% of Noonan syndrome cases and has a negative impact on prognosis.^2^, ^3^ Although septal myectomy is the gold standard treatment of drug‐refractory hypertrophic obstructive cardiomyopathy (HOCM),^4^ there are a few reports detailing the surgical outcome of HOCM in pediatric patients with Noonan syndrome.^5^, ^6^ We describe a 7‐year‐old boy with Noonan syndrome and an associated RAF1 mutation, with severe mitral regurgitation (MR) complicated by left ventricular outflow tract obstruction (LVOTO). He successfully underwent concomitant septal myectomy and mitral valve repair.

## 2 CASE REPORT

**FIGURE 1:**
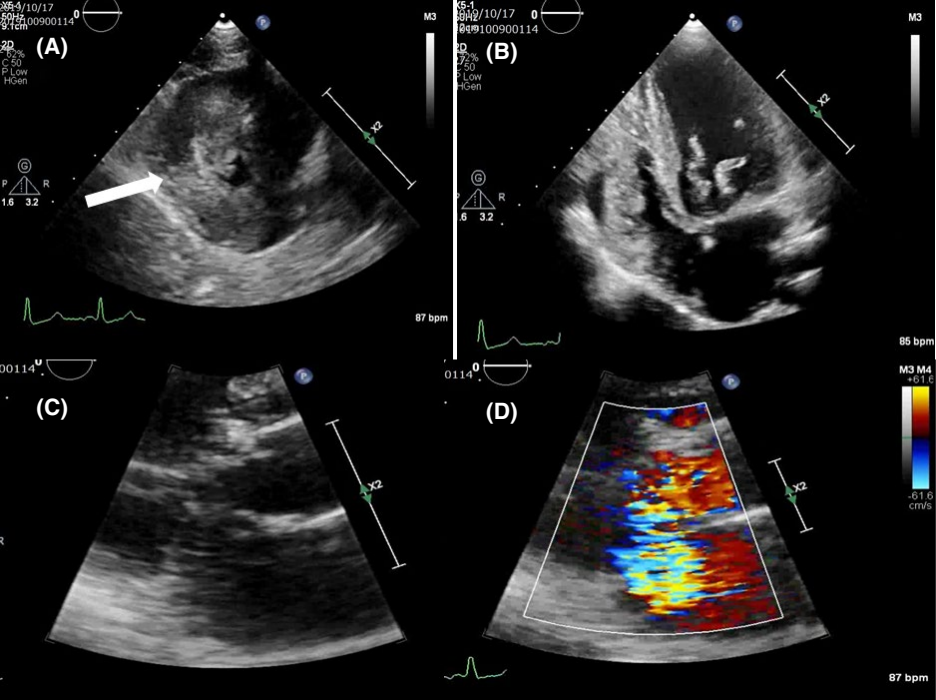
Preoperative transthoracic echocardiography images. A, The parasternal short‐axis view shows severe left ventricular hypertrophy (arrow). B, The apical 4‐chamber view shows degeneration and elongation of the mitral valve bileaflets. C, The B‐mode demonstrates systolic anterior motion of the mitral valve. D, A color Doppler demonstrates severe mitral regurgitation due to systolic anterior motion

The patient's family provided a written informed consent for publication of this report. The requirement for formal approval by the Ethics Committee was waived. A 7‐year‐old boy with Noonan syndrome presented to our hospital for surgery. The patient was diagnosed with Noonan syndrome at birth. He had idiosyncratic facial features, global developmental delay, and a RAF1 mutation. His condition was complicated with HOCM and treated with bisoprolol and cibenzoline. Despite continuation of optimal drug treatment, the LVOTO gradually worsened and became complicated by MR. Accordingly, the patient became aware of easy fatigue. Preoperative transthoracic echocardiography showed marked thickening of the left ventricular myocardium (septal thickness 21 mm), severe LVOTO (pressure gradient 64 mm Hg), and severe MR associated with systolic anterior motion (SAM) (Figure 1). We did not perform cardiac magnetic resonance imaging because echocardiography provided an adequate preoperative assessment. Clinical and laboratory findings revealed worsening symptoms and presence of severe LVOTO with a pressure gradient ≥50 mm Hg. Therefore, our multidisciplinary heart team determined that the patient was amenable to invasive treatment. Percutaneous alcohol septal ablation and dual chamber pacing therapy have insufficient evidence of safety and effectiveness, and surgical septal myectomy is currently the golden standard invasive treatment of this disease, especially in pediatric cases. The patient was considered to be sufficiently tolerant of open heart surgery. Consequently, we decided to perform a surgical septal myectomy rather than percutaneous alcohol septal ablation or dual chamber pacing therapy. The patient did not have a history of unexpected syncope, nonsustained ventricular tachycardia, or family history of sudden death. He was not considered to be at a high risk for sudden cardiac death. Thus, there was no need for implantable cardioverter defibrillator.

**FIGURE 2:**
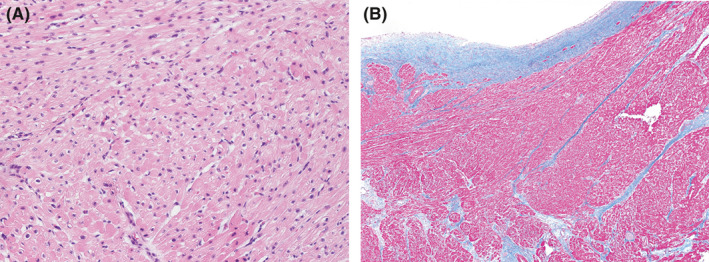
Histopathological images. A, Hypertrophy and intricate arrangement of the myocardium (Hematoxylin‐Eosin Stain, ×200). B, Fibrosis between the myocardial cells and severe endocardial fibroelastosis (Azan stain, ×40)

**FIGURE 3:**
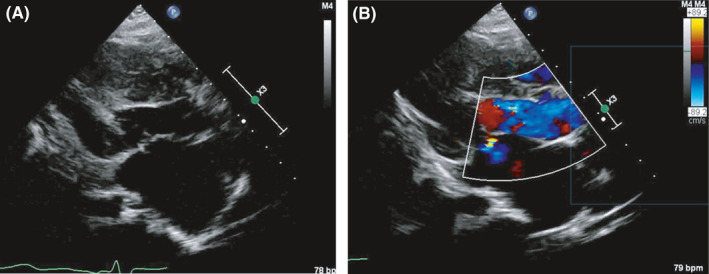
Postoperative transthoracic echocardiography images. A, The parasternal short‐axis view shows improvement in the left ventricular outflow tract obstruction and systolic anterior motion. B, A Color Doppler shows only a trace mitral regurgitation

The procedure was performed through a full median sternotomy. An extracorporeal circulation system was established through standard ascending aortic and bicaval cannulation. An aortic clamp and antegrade infusion of blood cardioplegia were used to arrest the heart. We incised the ascending aorta and observed the left ventricular outflow tract (LVOT) through the aortic valve. The septum below the aortic valve was markedly thickened and a 10 × 15 × 5 mm ventricular septum between the nadir of the right coronary cusp and the left/right commissure was resected with a scalpel according to Morrow's procedure. After this procedure, we found that the LVOT was sufficiently enlarged to allow the mitral valve leaflets and papillary muscles to be visible. Next, we created a left atrial incision. Both mitral valve leaflets were moderately degenerated with an elongated valve length. The mitral anterior leaflet was sutured and fixed to the relative posterior annulus near both fibrous trigons using two 5‐0 polypropylene sutures with autologous pericardial pledges as previously described by Hetzer et al^7^ Transesophageal echocardiography revealed an enlarged LVOT and a reduction in SAM and MR. Histological examination of the resected myocardium showed characteristic findings of hypertrophic cardiomyopathy, such as hypertrophy, intricate arrangement of the myocardial cells, fibrosis between the myocardial cells, and severe endocardial fibroelastosis (Figure 2). The patient's postoperative course was complicated by atrial fibrillation and ventricular tachycardia that were difficult to treat. However, his condition stabilized with the use of amiodarone. A postoperative transthoracic echocardiograph revealed a significant decrease in the LVOT gradient (pressure gradient 7 mm Hg), resolution of SAM, and a reduction in the MR grade (Figure 3). The patient was able to walk on discharge, 47 days after surgery.

## 3 DISCUSSION

Noonan syndrome is strongly associated with cardiac disease. A RAF1 mutation is known to increase the rate of hypertrophic cardiomyopathy,^1^ and Noonan syndrome has been reported to have a poor prognosis when complicated with HOCM.^3^ Our patient presented with severe LVOTO, MR due to SAM, and signs of heart failure. Therefore, his prognosis was considered to be poor and surgery was performed to increase his chances of recovery.

Generally, a transaortic septal myectomy is performed to treat HOCM, with good outcomes reported in the literature.^4^ Septal myectomy has also been reported to lead to good results in cases of Noonan syndrome accompanied by HOCM.^5^, ^6^ For MR associated with SAM and complicated by HOCM, a septal myectomy alone is often reported to improve symptoms if the mitral valve or mitral subvalvular apparatus has not been involved.^8^, ^9^ However, in the presence the mitral valve lesions, a septal myectomy alone may result in residual postoperative SAM and MR, leading to poor outcomes. Poterucha et al reported that SAM improved and MR was well controlled by septal myectomy alone in patients without Noonan syndrome. However, 25% of patients with Noonan syndrome required additional mitral valve interventions.^6^ Little has been reported on the relationship between Noonan syndrome and mitral valve disease. Yet, the possibility that pathological degeneration is more acute compared to those without Noonan syndrome cannot be ruled out.^10^ Therefore, in patients with Noonan syndrome, the intraoperative mitral valve findings should be thoroughly analyzed to determine whether additional mitral valve surgery should be performed. Delmo Walter et al reported that if additional mitral valve surgery was required, mitral valve leaflet retention repair would be simple and have good outcomes.^11^ The complex mitral valve repair procedure is more difficult to perform in children than in adults. In this respect, retention repair is an easy and reproducible procedure and considered very effective in pediatric cases.

## 4 CONCLUSION

We performed a septal myectomy and a mitral valve leaflet retention repair in a boy with Noonan syndrome and a RAF1 mutation who had HOCM and MR. This case shows that a septal myectomy alone may improve SAM‐related MR. However, a concomitant mitral valve leaflet retention repair could improve both postoperative SAM and MR if the mitral valve itself is degenerated due to Noonan syndrome‐associated hypertrophic cardiomyopathy, potentially reducing the number of additional surgeries that may be required in the future.

## CONFLICT OF INTEREST

The authors have no conflicts of interest to declare.

**AUTHOR CONTRIBUTIONS**

TD: contributed to data collection, manuscript drafting and preparation. DT, AH, SY, and KF: contributed to data analysis and revision of the manuscript. NY: contributed to final editing of the manuscript.

## ETHICAL APPROVAL

The data that support the findings of this study are available on request from the corresponding author. The data are not publicly available due to privacy or ethical restrictions.
